# Endoplasmic reticulum and inner nuclear membrane ubiquitin-conjugating enzymes Ubc6 and Ubc7 confer resistance to hygromycin B in *Saccharomyces cerevisiae*

**DOI:** 10.17912/micropub.biology.001276

**Published:** 2024-07-29

**Authors:** Sophia L Owutey, Katrina A Procuniar, Emmanuel Akoto, Jacob C Davis, Rachel M Vachon, LiLi F O'Malley, Hayden O Schneider, Philip J Smaldino, Jason D True, Ashley L Kalinski, Eric M Rubenstein

**Affiliations:** 1 Department of Biology, Ball State University; 2 Department of Anesthesiology, University of North Carolina; 3 Division of Molecular Cardiovascular Biology, Cincinnati Children’s Hospital Medical Center

## Abstract

Aberrant endoplasmic reticulum (ER) and inner nuclear membrane (INM) proteins are destroyed through ER-associated degradation (ERAD) and INM-associated degradation (INMAD). We previously showed the Hrd1, Doa10, and Asi ERAD and INMAD ubiquitin ligases (E3s) in *Saccharomyces cerevisiae* confer resistance to hygromycin B, which distorts the ribosome decoding center. Here, we assessed the requirement of Ubc6 and Ubc7, the primary ERAD and INMAD ubiquitin-conjugating enzymes (E2s) for hygromycin B resistance. Loss of either E2 sensitized cells to hygromycin B, with *UBC7* deletion having a greater impact, consistent with characterized roles for Ubc6 and Ubc7 in ER and INM protein quality control.

**Figure 1. UBC6 and UBC7 confer resistance to hygromycin B:**
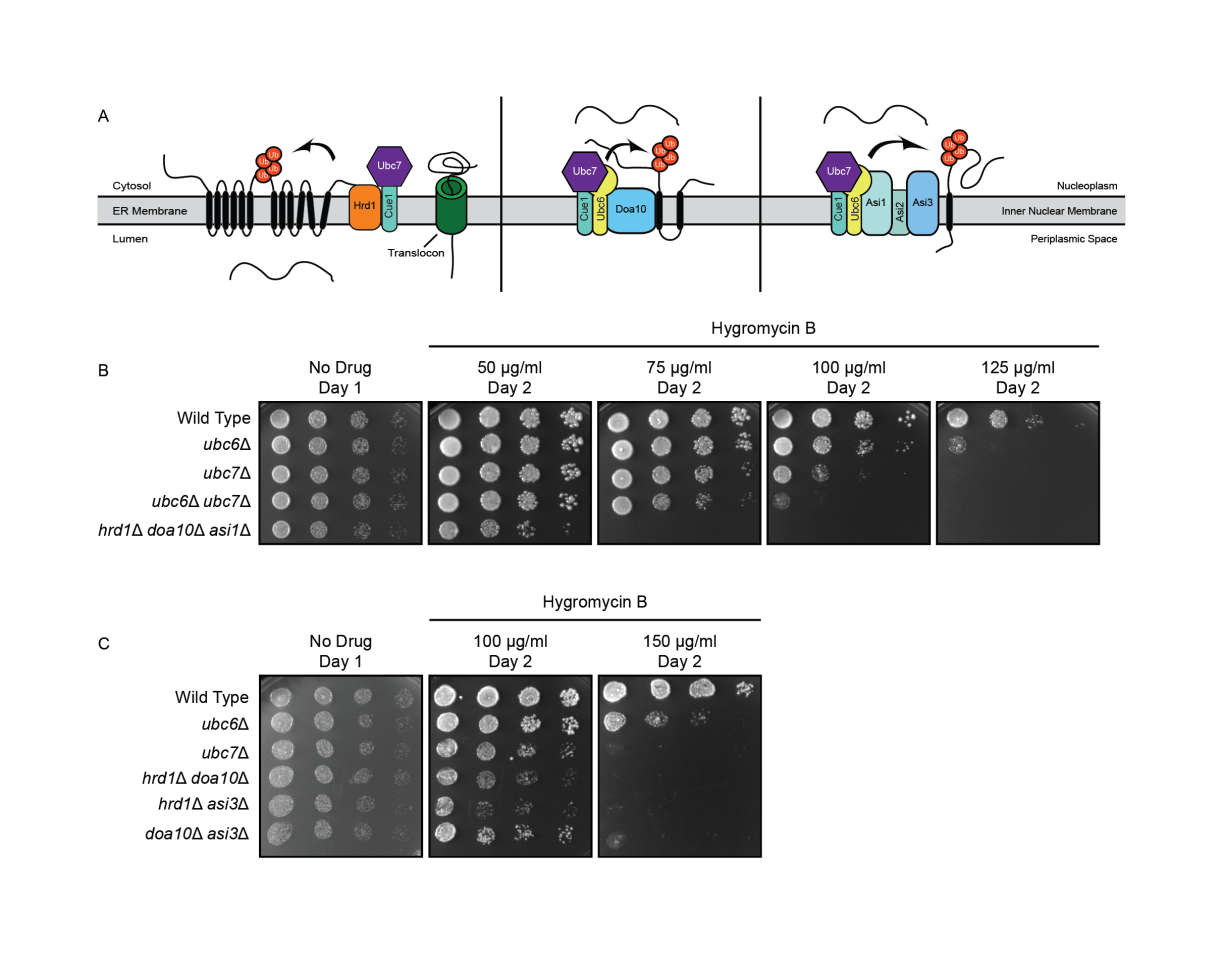
**(A)** Endoplasmic Reticulum (ER)-Associated Degradation and Inner Nuclear Membrane (INM)-Associated Degradation pathways. In conjunction with the E2 Ubc7, the E3 Hrd1 promotes degradation of aberrant ER luminal and transmembrane proteins as well as proteins that clog ER translocons. The E3 Doa10 functions with two E2s, Ubc6 and Ubc7, to mediate degradation of aberrant transmembrane proteins at the ER or INM in addition to soluble cytosolic or nucleoplasmic proteins. The trimeric Asi E3 complex (Asi1, Asi2, and Asi3) works with Ubc6 and Ubc7 to target aberrant transmembrane INM and soluble nucleoplasmic proteins. Ubc7 is anchored at the ER membrane through interaction with Cue1. Ub, ubiquitin. **(B)** and **(C)** Sixfold serial dilutions of yeast of the indicated genotype were spotted on medium lacking (No Drug) or containing increasing concentrations of hygromycin B. Plates were incubated at 30°C and imaged after 1-2 days. Experiments were performed three or more times.

## Description

Degradation of misfolded, excess, and otherwise aberrant proteins is critical for cellular homeostasis. The ability to recognize and destroy faulty proteins declines with age, and disruptions to enzymes contributing to protein quality control (PQC) contribute to several diseases (Badawi et al., 2023; Guerriero & Brodsky, 2012) . Eukaryotic cells possess compartment-specific PQC mechanisms, including those dedicated to the turnover of aberrant proteins at the physically continuous endoplasmic reticulum (ER) membrane and inner nuclear membrane (INM) (Mehrtash & Hochstrasser, 2019) . ER-associated degradation (ERAD) promotes turnover of aberrant ER luminal, transmembrane, and translocon-clogging proteins as well as cytosolic polypeptides that contact the ER surface. INM-associated degradation (INMAD) mediates proteolysis of faulty INM transmembrane and INM-abutting soluble nucleoplasmic proteins. ERAD and INMAD both employ ubiquitin-conjugating enzymes (E2s) and ubiquitin ligases (E3s) to polyubiquitylate proteins ( Figure 1A ), destining them for destruction by cytosolic or nucleoplasmic proteasomes.

The highly conserved transmembrane Hrd1 and Doa10 E3s mediate ERAD in *Saccharomyces cerevisiae* , targeting distinct classes of aberrant proteins for degradation based on the location and nature of the degradation signals (degrons) (Carvalho et al., 2006; Huyer et al., 2004; Metzger et al., 2008; Rubenstein et al., 2012; Runnebohm, Richards, et al., 2020; Sato et al., 2009) . Doa10 also functions in INMAD alongside the heterotrimeric Asi E3 (composed of Asi1, Asi2, and Asi3) (Deng & Hochstrasser, 2006; Foresti et al., 2014; Khmelinskii et al., 2014) . Loss of either Asi1 or Asi3 abolishes Asi PQC function (Foresti et al., 2014; Woodruff et al., 2021) . Hrd1, Doa10, and the Asi complex have partially overlapping E2 dependencies. Hrd1 functions primarily with the soluble E2 Ubc7 (human homolog, UBE2G2), which is anchored at the membrane by the transmembrane protein Cue1 (Bays et al., 2001; Lips et al., 2020; Plemper et al., 1999) . By contrast, Doa10 and Asi use two E2s, Ubc7 and the transmembrane Ubc6 (human homolog, UBE2J2) (Foresti et al., 2014; Khmelinskii et al., 2014; Swanson et al., 2001) . Ubc6 and Ubc7 participate in a sequential ubiquitylation mechanism, with Ubc6 “priming” substrates with an initial ubiquitin molecule and Ubc7 elongating polyubiquitin chains (Lips et al., 2020; Weber et al., 2016) . It is likely that additional E2s contribute to a lesser extent to ERAD and INMAD. For example, in some circumstances, the E2 Ubc1 partially compensates for impaired Ubc7 function in promoting Hrd1 substrate ubiquitylation (Bays et al., 2001) .

The aminoglycoside hygromycin B binds to and distorts the ribosome A site, thereby likely increasing the frequency of mistranslation and generation of PQC substrates (Brodersen et al., 2000; Ganoza & Kiel, 2001) . Mutation of genes encoding several proteins with documented or predicted PQC function causes hygromycin B hypersensitivity (Bengtson & Joazeiro, 2010; Chuang & Madura, 2005; Daraghmi et al., 2023; Flagg et al., 2023; Jaeger et al., 2018; Turk et al., 2023; Verma et al., 2013) . Indeed, we have previously shown that loss of several ubiquitin ligases, including Hrd1, Doa10, Asi1, or Asi3, sensitizes cells to hygromycin B (Crowder et al., 2015; Doss et al., 2022; Niekamp et al., 2019; Runnebohm, Evans, et al., 2020; Woodruff et al., 2021) . A role for Ubc6 and Ubc7 in combatting hygromycin B-induced proteotoxic stress has not been demonstrated. Given their functions as the major characterized E2s in ERAD and INMAD, we predicted loss of either enzyme would reduce fitness in the presence of this drug.

To assess the roles of Ubc6 and Ubc7 in combatting proteotoxic stress caused by hygromycin B, we cultured serial dilutions of wild type yeast, yeast lacking *UBC6* and *UBC7* individually or in concert, as well as a yeast strain rendered broadly defective for ERAD and INMAD by simultaneous deletion of *HRD1* , *DOA10* , and *ASI1* ( Figure 1B ). All strains grew similarly in the absence of hygromycin B. Loss of either *UBC6* or *UBC7* sensitized yeast to hygromycin B, with *UBC7* deletion having a stronger impact. Combined deletion of both *UBC6* and *UBC7* caused a greater growth defect than individual absence of either E2-encoding gene. Finally, *hrd1* Δ *doa10* Δ *asi1* Δ yeast exhibited a more profound growth defect than any E2 mutant.

To validate these results, we assessed hygromycin B sensitivity of *ubc6* Δ and *ubc7* Δ yeast strains in a distinct genetic background, as well as three double mutants lacking catalytic components of the ERAD or INMAD E3s ( Figure 1C ). As before, loss of either E2 sensitized yeast to hygromycin B, with cells lacking Ubc7 faring more poorly than those without Ubc6. Loss of any two ERAD or INMAD E3s approximately phenocopied *ubc7* Δ yeast.

A greater role for Ubc7 than Ubc6 in combatting proteotoxicity likely reflects broader Ubc7 participation in ERAD and INMAD. Loss of Ubc6 is expected to compromise Doa10 and Asi function, while *UBC7* deletion is predicted to abolish all three major branches of ERAD and INMAD. The observation that *ubc6* Δ *ubc7* Δ double mutant yeast exhibit a stronger growth defect than either *ubc6* Δ or *ubc7* Δ single mutant suggests independent functions for both Ubc6 and Ubc7. Identification of Ubc6-dependent, Ubc7-independent PQC substrates would support this model. Further, an enhanced growth defect of *hrd1* Δ *doa10* Δ *asi1* Δ compared to *ubc6* Δ *ubc7* Δ yeast is in agreement with other reports indicating additional E2s (such as Ubc1) may function with ERAD and INMAD E3s, when the primary E2s are unavailable.

Our data are consistent with a previous study demonstrating overexpression of genes encoding either E2 enhances resistence to multiple stresses, including heat stress, oxidative stress, and presence of the toxic amino acid analog canavanine (Hiraishi et al., 2006) . Conversely, previous work showed that *ubc7* Δ and *hrd1* Δ *doa10* Δ yeast exhibited similar hypersensitivity to cadmium (Swanson et al., 2001) . Large-scale analyses indicated loss of *UBC7* reduces tolerance to multiple transition metals, which oxidatively damage a range of biological macromolecules, including proteins (Bleackley et al., 2011; Ruotolo et al., 2008; Zhao et al., 2020) , and genotoxic agents (Alamgir et al., 2010; Brown et al., 2006; Gaytan et al., 2013; Kapitzky et al., 2010) . We note hygromycin B hypersensitivity was not observed for *ubc6* Δ or *ubc7* Δ yeast in a previous report (Chuang & Madura, 2005) . This may be due to differences in effective drug concentrations in culture medium. In alignment with our results, we have also recently shown that loss of Doa10, Hrd1, and Ubc7 homologs sensitizes the pathogenic fungi *Candida albicans* to hygromycin B (Doss et al., 2023) . Overall, our work supports a critical and conserved function for endoplasmic reticulum and inner nuclear membrane ubiquitin-conjugating enzymes in protein quality control.

## Methods

**Growth assays**

Yeast growth was analyzed as previously described (Watts et al., 2015) . Briefly, sixfold dilutions of each yeast strain were spotted onto yeast extract-peptone-dextrose medium (Guthrie & Fink, 2004) lacking or containing hygromycin B (Gibco) at the indicated concentrations and incubated at 30°C for the indicated amount of time.

***ASI3* gene replacement**

To generate yeast strains VJY409 and VJY410, *ASI3* was replaced by *natMX4* through homologous recombination. A 1464-bp *nat4MX4* cassette with termini possessing sequences flanking the *ASI3* gene was PCR-amplified from pAG25 (Goldstein & McCusker, 1999) using primers VJR274 (5’ AGGAACAGTCATTACGTAGGGATTTTCAAAAGTTTGACTGCACATACGATTTAGGTGACAC) and VJR275 (5’ TCCTATGATGTCTTAAATACGTATACCTAATAAAATAATTAATACGACTCACTATAGGGAG 3’). The *natMX4* cassette was introduced into VJY22 ( *hrd1* Δ *::kanMX4* ) yeast and VJY102 ( *doa10* Δ *::kanMX4* ) by lithium acetate transformation (Guthrie & Fink, 2004) . Successful integration in nourseothricin-resistant clones were verified by PCR at the 5’ and 3’ recombination junctions, and genotypes at the *DOA10* , *HRD1* , and *ASI3* loci were PCR validated for both strains.

## Reagents

***Yeast strains used in this study.***

**Table:** 

**Name**	**Genotype**	**Figure or purpose**	**Reference**
VJY6 (alias MHY500)	*MATa his3-* Δ *200 leu2-3,112 ura3-52 lys2-801 trp1-1 gal2*	1B	(Chen et al., 1993)
VJY22	*MATa his3* Δ *1 leu2* Δ *0 met15* Δ *0 ura3Δ0 hrd1* Δ:: *kanMX4*	Used to generate VJY409	(Tong et al., 2001)
VJY44 (alias MHY496)	*MATa his3-* Δ *200 leu2-3,112 ura3-52 lys2-801 trp1-1 gal2 ubc6-* Δ *1* :: *HIS3*	1B	(Sommer & Jentsch, 1993)
VJY50 (alias MHY551)	*MATa his3-* Δ *200 leu2-3,112 ura3-52 lys2-801 trp1-1 gal2 ubc7* Δ:: *LEU2*	1B	(Chen et al., 1993)
VJY102	*MATa his3* Δ *1 leu2* Δ *0 met15* Δ *0 ura3* Δ *0 doa10* Δ:: *kanMX4*	Used to generate VJY410	(Tong et al., 2001)
VJY305 (alias SKY252)	*MATa his3* Δ *1 leu2* Δ *0 met15* Δ *0 ura3* Δ *0 doa10* Δ:: *kanMX4 hrd1* Δ:: *kanMX4*	1C	(Habeck et al., 2015)
VJY409	*MATa his3* Δ *1 leu2* Δ *0 met15* Δ *0 ura3* Δ *0 hrd1* Δ:: *kanMX4 asi3* Δ:: *natMX4*	1C	This study
VJY410	*MATa his3* Δ *1 leu2* Δ *0 met15* Δ *0 ura3* Δ *0 doa10* Δ:: *kanMX4 asi3* Δ:: *natMX4*	1C	This study
VJY476 (alias BY4741)	*MATa his3* Δ *1 leu2* Δ *0 met15* Δ *0 ura3* Δ *0*	1C	(Tong et al., 2001)
VJY723	*MATa his3* Δ *1 leu2* Δ *0 met15* Δ *0 ura3* Δ *0 ubc6* Δ:: *kanMX4*	1C	(Hickey et al., 2021)
VJY1075	*MATa his3* Δ *1 leu2* Δ *0 met15* Δ *0 ura3* Δ *0 ubc7* Δ:: *kanMX4*	1C	(Tong et al., 2001)
VJY1096 (alias MHY553)	*MATa his3-* Δ *200 leu2-3,112 ura3-52 lys2-801 trp1-1 gal2 ubc6* Δ:: *HIS3 ubc7* Δ:: *LEU2*	1B	(Chen et al., 1993)
VJY1098 (MHY11132, ABM297)	*MATa his3-* Δ *200 leu2-3,112 ura3-52 lys2-801 trp1-1 gal2 doa10* Δ:: *HIS3 hrd1* Δ:: *LEU2 asi1* Δ:: *kanMX6*	1B	(Mehrtash & Hochstrasser, 2023)
